# A Population Dynamics Analysis of the Interaction between Adaptive Regulatory T Cells and Antigen Presenting Cells

**DOI:** 10.1371/journal.pone.0002306

**Published:** 2008-05-28

**Authors:** David Fouchet, Roland Regoes

**Affiliations:** Institute of Integrative Biology, Die Eidgenössische Technische Hochschule (ETH) Zürich, Die Eidgenössische Technische Hochschule (ETH) Zentrum, Zürich, Switzerland; University of Sheffield, United Kingdom

## Abstract

**Background:**

Regulatory T cells are central actors in the maintenance of tolerance of self-antigens or allergens and in the regulation of the intensity of the immune response during infections by pathogens. An understanding of the network of the interaction between regulatory T cells, antigen presenting cells and effector T cells is starting to emerge. Dynamical systems analysis can help to understand the dynamical properties of an interaction network and can shed light on the different tasks that can be accomplished by a network.

**Methodology and Principal Findings:**

We used a mathematical model to describe a interaction network of adaptive regulatory T cells, in which mature precursor T cells may differentiate into either adaptive regulatory T cells or effector T cells, depending on the activation state of the cell by which the antigen was presented. Using an equilibrium analysis of the mathematical model we show that, for some parameters, the network has two stable equilibrium states: one in which effector T cells are strongly regulated by regulatory T cells and another in which effector T cells are not regulated because the regulatory T cell population is vanishingly small. We then simulate different types of perturbations, such as the introduction of an antigen into a virgin system, and look at the state into which the system falls. We find that whether or not the interaction network switches from the regulated (tolerant) state to the unregulated state depends on the strength of the antigenic stimulus and the state from which the network has been perturbed.

**Conclusion/Significance:**

Our findings suggest that the interaction network studied in this paper plays an essential part in generating and maintaining tolerance against allergens and self-antigens.

## Introduction

Developing an adequate immune response against antigens is vital for all animal species. To respond adequately, an immune system must discriminate harmful foreign pathogens from beneficial microbes and self-antigens. An important player in balancing benefits and costs of immune responses are regulatory T cells. They are involved in the control of auto-immunity, the induction of tolerance to foreign antigens, but also in limiting immunopathology during both acute and chronic infections.

The past decade has seen the large expansion of the characterization of regulatory T cells [1] and their classification into distinct subsets. The two main types of regulatory T cells are the so-called natural and adaptive regulatory T cells [2], [3]. While natural regulatory T cells are produced by the thymus and are committed suppressors of immunity from the beginning of their life [4], adaptive regulatory T cells can be induced in the periphery from precursor T cells [5], [6] that could otherwise turn into effector cells.

**Figure 1:**
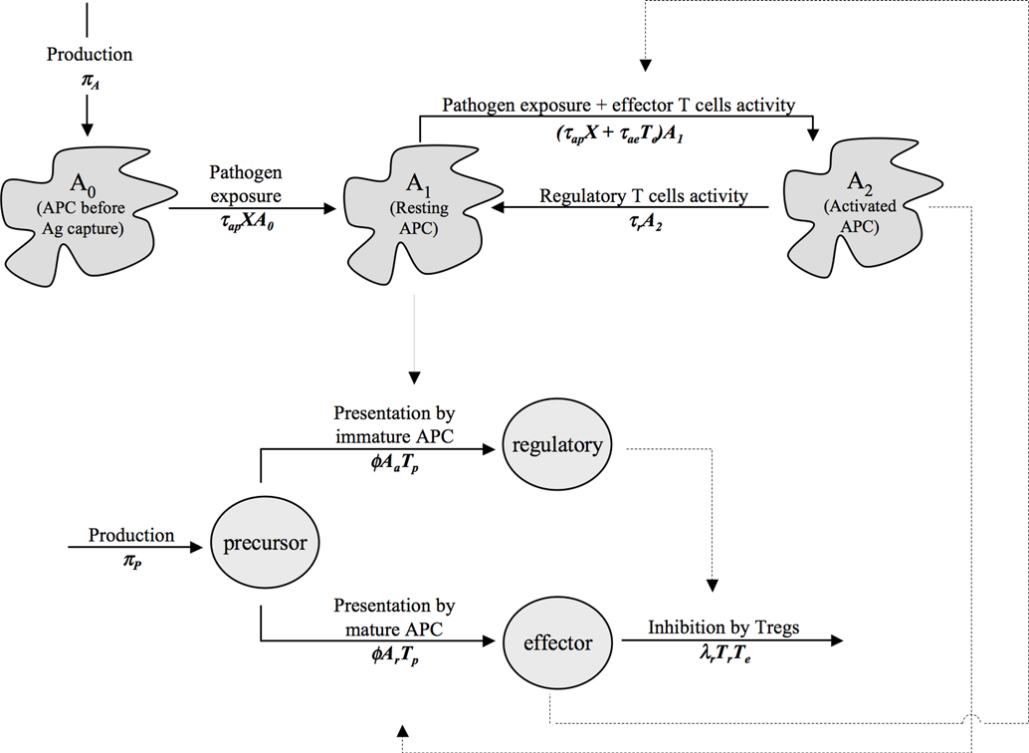
Interaction network of the immune response. Solid arrows describe the evolution of the different cell types (APCs or lymphocyte T CD4+ cells). Dashed arrows indicate the cell types involved in the changes (for example regulatory T cells are involved in the inhibition of effector T cells). The mathematical model developed in this paper is based on this interaction network. Rates of the mathematical model are recalled under the evolution arrows. To make it clearer, we omitted the death of all cell types in the Figure. The antigen *X* is not represented here. Note that in the model proposed by Powrie and Maloy (2003), the activity of activated APCs can revert the inhibition of effector T cells by regulatory T cells. For the sake of simplicity, we omitted this interaction here. This does not deeply affect the qualitative nature of the results (results not shown).

How adaptive regulatory T cells are regulated is still under debate, but experimental evidence converges toward some important features. The interaction network proposed by Powrie and Maloy [7] (represented schematically in Fig. 1) summarizes one route by which adaptive regulatory T cells can be induced.

The differentiation of mature T cells into effector cells requires the presentation of the antigen by antigen presenting cells (APC) presenting the appropriate co-stimulatory signals [8], [9], such as a high level of expression of the B7 co-receptors (CD80 and CD86). Without the appropriate cosignal, T cells will differentiate into regulatory T cells [10]–[12]. This dichotomy is illustrated by the experiments conducted by Jonuleit et al. [13], who stimulated naive, allogeneic CD4(+) T cells with immature CD83(−) and mature CD83(+) human dendritic cells (DC, a class of professional APCs). They showed mature DCs induced inflammatory Th1 cells whereas immature DCs induced IL-10-producing T cell regulatory 1-like.

In return, each T cell type will promote the activation of APCs in a way that favors its production. Effector cells favors the activation of DCs through the CD40-CD40L interaction [14]. Conversely, several experiments have shown that DCs treated with IL-10, a cytokine secreted by regulatory T cells such as type 1 regulatory T cells, are rendered immature and induce the differentiation of CD4(+) cells into IL-10 producing regulatory T cells [15]–[17]. The last important actor is the antigen that activates the APCs, either directly, via Toll-like receptors of the APC, or indirectly, by triggering the secretion of pro-inflammatory cytokines.

Regulatory T cells ensure several different functions. They are involved in the control of auto-immunity as well as in the regulation of both acute and chronic infections. It would be surprising that one interaction network alone could ensure all these functions. Using a mathematical model, we study how the immune system responds to different degrees of antigenic stimulation. Our results suggest that the induction of regulatory T cells by resting APCs may play an important role in the prevention of auto-immune diseases.

## Methods

The mathematical model is based on the interaction network proposed by Powrie and Maloy [7] (see Fig. 1). Two cell lineages are represented: Antigen Presenting Cells (APC, e.g. Dendritic Cells) and CD4+ lymphocyte T cells. We define as *A_0_* the APCs that have not captured the antigen, as resting (*A_1_*) the APCs that have captured the antigen and that induce precursor cells to differentiate into regulatory T cells upon contact and as activated (*A_2_*) the APCs that have captured the antigen and that induce precursor cells to differentiate into effector cells upon contact. T cells are ranged into three classes: precursor (*T_p_*, i.e. mature but not yet activated by the APC), effector (*T_e_*, i.e. cells that will control the pathogen spread) and regulatory T cells (*T_r_*).

The antigen is denoted by *X*. It can be e.g. a pathogen agent, a self-peptide or an allergen. Different antigens have different dynamics, and here we use an overly simplified one. Antigens are produced with a constant rate π*_X_*, die with a rate *m_X_* and are killed by effector cells with a rate *k*.

Resting APCs are produced with a constant rate π*_A_* and die with a rate *m_A_*. They capture the antigen with a rate τ*_ap_*, called the antigenic stimulation. To simplify we assume that antigens induce the activation of the APCs with the same rate. Effector cells also induce APC activation with a rate τ*_ae_*. Activation is reversed by regulatory T cells activity with a rate τ*_r_*.

We assume a constant influx (π*_P_*) of precursor T cells from the thymus. Then, depending on the first APC that the cell will meet, it will acquire either an effector or a regulatory phenotype. To simplify, we assume that all APCs are equally “attractive”, i.e. the rate of differentiation of precursor cells (φ) per APC is the same whatever the level of activation of the APC. Effector cells are inhibited by regulatory T cells with a rate λ*_r_*. We call *m_p_* the mortality rate of precursor cells. Effector and regulatory T cells have more complex dynamics. They can divide or die depending on the cytokines they receive. Modeling these complex dynamic is not the purpose here, so to simplify we just assume that the effector and regulatory T cells populations decline with given rates called turnover rates (respectively *m_e_* and *m_r_*) in absence of newly differentiated precursor cells. In the equilibrium, and in absence of inhibition of effector T cells by regulatory T cells, this assumption leads to a number of effector and regulatory T cells that are proportional to the frequency of activated and resting APCs, respectively. The model then reads:
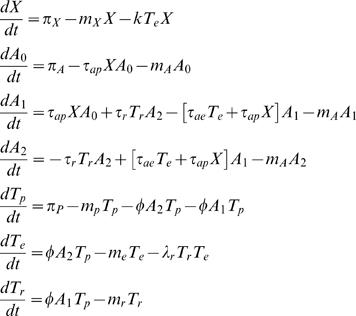



Note that in the interaction network proposed by Powrie and Maloy [7], activated APCs may revert the inhibitory effect of regulatory T cells on effector T cells [18]. For the sake of simplicity, we did not include this phenomenon here.

The model being too complex to be treated analytically, we use numerical simulations to estimate the dynamic of the system and its equilibria. This requires to estimate the value of the parameters. Determining accurate values is unfortunately impossible. First, the model does not focus on a specific species. Many parameters differ between species and so have no standard value. Second, many parameters are difficult to determine. For example, if it is clear that regulatory T cells may revert the activation of APCs, it is not known how much regulatory T cells are required for the reversion of one APC.

The approach we use here consists in scanning vast ranges of possible parameters and investigating the dynamical properties of the system. We are confident that we did not miss any dynamical behavior the model can display. This strategy of scanning parameter space is a more comprehensive way to look at a dynamical system that a strategy based on plausible parameterization. To sum up we focus on the dynamics and generic principles involved in the regulation of the immune response, rather than on a numerically accurate description of such systems.

Basic values of the parameters are given in Table 1. In the present paper we only show the impact of the most significative ones. Basic parameters are chosen according to the following rules. The antigens and APCs are normalized: their maximum value is one. This is obtained by setting their production rate equal to they death rate. In optimal condition, where all APCs are activated and regulatory T cells are depleted, 99% of the antigen is depleted. We assume that one antigen is produced (or introduced) per day, but this value is not critical since most of the study focuses on the equilibrium.

**Table 1:** Basic value of the parameters (time unit is the day).

Parameter	Symbol	Relation	Basic value
Reproduction rate of the antigen	π*_X_*		1
Death rate of the antigen	*m_X_*		1
Birth rate of precursor cells	π*_P_*		1
Mortality rate of precursor cells	*m_p_*		10^−2^
Rate of effector T cells decay	*m_e_*		0.1
Rate of regulatory T cells decay	*m_r_*		0.1
Birth rate of APCs	π*_A_*		0.2
Death rate of APCs	*m_A_*		0.2
Maximum number of effector T cells	*T_e_^max^*	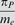	10
Maximum number of regulatory T cells	*T_r_^max^*	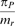	10
Rate of pathogen killing by effector cells	*k*	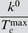	*k^0^* = 10^2^
Rate of APC activation by the antigen	τ*_ap_*		variable
Rate of APC reactivation by effector cells	τ*_ae_*	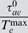	variable
Rate of differentiation of precursor cells by APCs	φ		1
Rate of inhibition of effector T cell by regulatory T cells	λ*_r_*	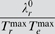	variable
Rate of APC inhibition by regulatory T cells	τ*_r_*	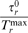	variable

In absence of antigen, hundred precursor cells (that show the specificity of the antigen) pre-exist. They have a 1% turnover per day. We assume that when all APCs have captured the antigen, 99% of the precursor cells have differentiated (into effector cells or regulatory T cells).

The maximum number of regulatory T cells (i.e. when all APCs are resting) is amongst the parameters that are difficult to estimate. This is due to the wide variety of regulatory T cells, the lack of simple markers for adaptive regulatory T cells and the fact that the number of regulatory T cells that are observed in experiments also depends on the fraction of APCs that are resting. In fact, we do have to determine this parameter here. One can easily show that reducing the maximal number of regulatory T cells can be easily compensated by increasing the impact each regulatory T cells have on other cells types, through the formulas that are given in Table 1. The same principle applies to effector T cells.

Finally the turnover rates (*m_e_* and *m_r_*) are also among the parameters that are complex to estimate. In our model, these parameters do not just denote the death rates of effector and regulatory cells, but indirectly also their proliferation rates (more precisely the time required for a cell population to reach its equilibrium value is decreasing with its turnover rate). Thus, these parameters denote the turnover rates of effector and regulatory cells. Arbitrarily we fixed these two turnover rates to 0.1. In particular it means there is around 10 days between the introduction of the antigen and the peak in the effector T cell response. Regulatory and effector T cells turnover rates have no impact here on the equilibrium states. This is due to the compensation rules showed in Table 1.

## Results

### A) Equilibrium analysis

#### The dynamical system tends to extreme: strong or weak regulation

To investigate the role of regulatory T cells in balancing the benefits and costs of immune responses against chronic infection, allergens and self-antigens, we performed an equilibrium analysis of the population dynamical system described in the Method section and Fig. 1. An equilibrium analysis allows us to study the long-term behavior of population dynamical systems.

**Figure 2:**
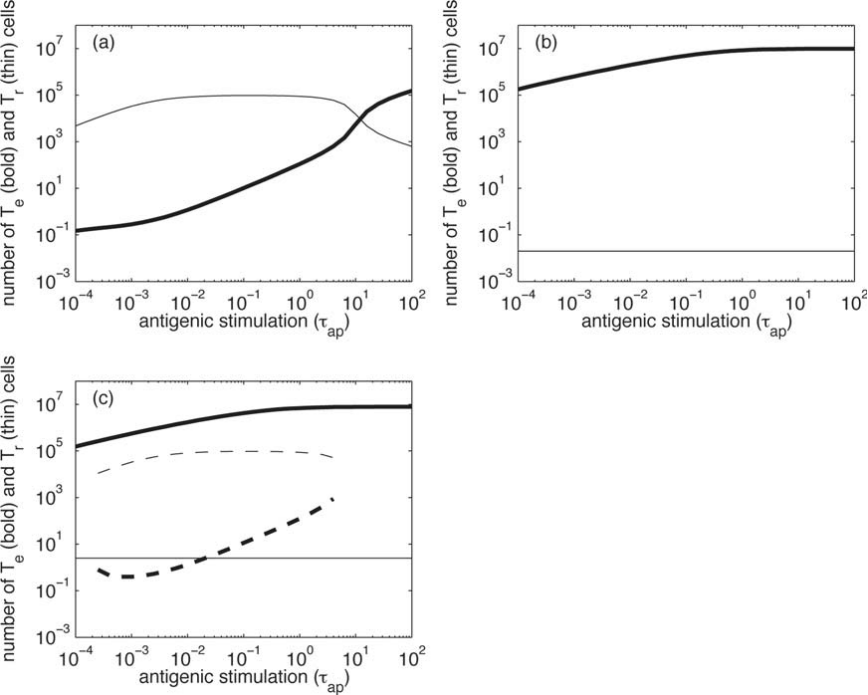
Equilibrium states of the system. Number of effector (bold lines) and regulatory (thin lines) cells in the equilibrium states according to the antigenic stimulation (τ*_ap_*), in: (a) an example of situation with only one stable and strongly regulated equilibrium (τ*_ae_^0^* = 10^2^); (b) an example of situation with only one stable and weakly regulated equilibrium (τ*_ae_^0^* = 10^6^) and (c) an example of bi-stable situation with an unstable equilibrium in between (τ*_ae_^0^* = 10^4^). To distinguish between the two equilibrium states, the strongly regulated one is plotted with dashed lines. Note also that only stable states are presented here. In the bistable regime there is always another equilibrium state that is unstable. λ*_r_^0^* = 10^4^, τ*_r_^0^* = 10 in all the situations.

The particular antagonistic nature of the interaction between regulatory and effector T cells in the model leads effectively to the competitive exclusion of effectors or regulatory T cells. The reason for this is that more regulatory T cells leads to more resting APCs, and finally to more regulatory T cells. Similarly, more effector cells lead to more activated APCs and thus to more effector cells. In such dynamical systems, one of the cell types will outcompete the other. This lead to either a state of weak regulation, in which effector T cells are abundant and the levels of regulatory T cells are very low, or to a strongly regulated state, in which effector cells are strongly repressed by regulatory cells. The suppression of one cell type by the other never leads to extinction because resting APCs are constantly produced by the capture of the antigen and activated APCs are constantly produced by antigenic stimulation. In the strongly regulated state, the number of effector cells is much lower than the level of effector cells in the weakly regulated state (Fig. 2). However, highly stimulating antigens can revert the suppression of effector cells. In any case, highly stimulating antigens always lead to a weakly regulated state (see Fig. 2). The nature of the state in which the system falls has direct consequences on the antigen. In the strongly regulated state the antigen is almost not suppressed by the immune system whereas in the weakly regulated the strong immune response that is mounted leads to a large reduction in the antigen level (results not shown).

We find that there are three parameter regimes:

1. Regime 1 in which there is only a strongly regulated state (Fig. 2a),
2. Regime 2 in which there is only a weakly regulated state (Fig. 2b), and
3. Regime 3 in which both, strongly and weakly regulated states, can be attained (Fig. 2c).

#### Conditions for bi-stability

We investigated the boundaries between these three parameter regimes. In the following analysis, we focus on three parameters: τ*_r_^0^*, λ*_r_^0^* and τ*_ae_^0^*. The first two rate constants describe the effect of regulatory T cells on APCs and effector T cells, respectively, and τ*_ae_^0^* describes the effect of effector T cells on APCs. These rate constants are at the center of the regulatory network that our model describes (see Fig. 1).

Regime 1 is characterized by a large impact of regulatory T cells (either on APCs or on effector cells) or a small impact of effector cells on APCs, while Regime 2 is characterized by a small impact of regulatory T cells and a large impact of effector cells. Regime 1 and 2 are separated by Regime 3.

**Figure 3:**
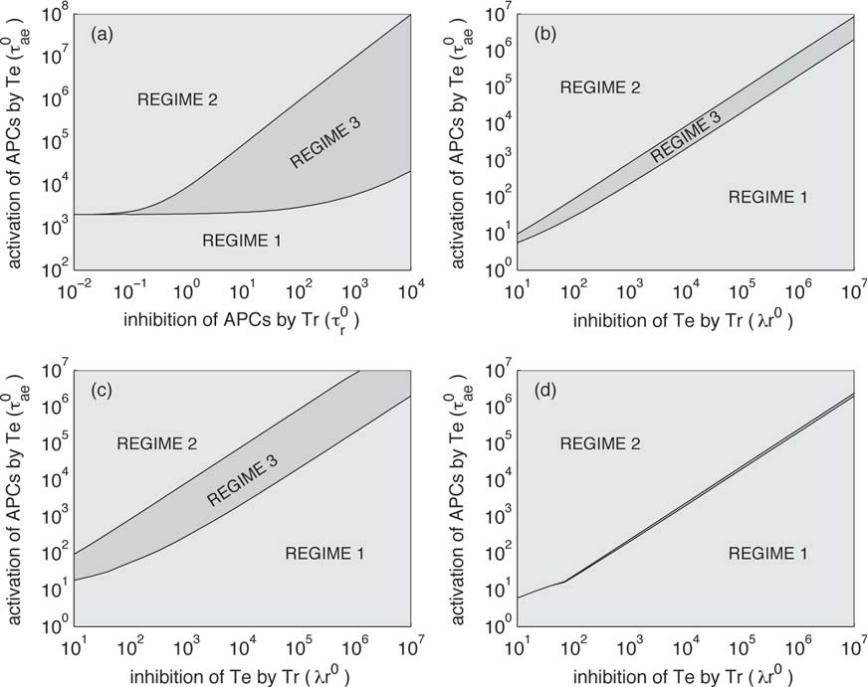
Impact of the parameters on the nature of the equilibrium regime. Situations are divided into the 3 regimes described in the main text (see also Fig. 2). (a) For different values of the activation rate of APCs by effector cells (τ*_ae_^0^*) and different rates of regression of APCs by regulatory T cells (τ*_r_^0^*), with λ*_r_^0^* = 10^4^; (b) for different values of the activation rate of APCs by effector T cells (τ*_ae_^0^*) and different rates of inhibition of effector cells by regulatory T cells (λ*_r_^0^*), with τ*_r_^0^* = 1; (c) same as (b) but with τ*_r_^0^* = 10; (d) same as (b) but with τ*_r_^0^* = 0.1.

Regime 3 in which bistability occurs becomes larger if the maximum inhibitory effect of regulatory T cells on APC activation state increases (parameter τ*_r_^0^*) (Fig. 3a). The size of the bistability region depends on how resting APC arise. The bistability region is large if resting APCs arise more frequently by de-activation of resting APCs by regulatory T cells (τ*_r_^0^*) than by naïve APCs that capture the pathogen (τ*_ap_X*). If regulatory T cells do not affect activated APCs bi-stability is not predicted by our model.

Fig. 3b–d show how the bistability region depends on the λ*_r_^0^* and τ*_ae_^0^*, i.e. the rates constants describing the inhibition of effector T cells by regulatory T cells and APC activation induced by effector T cells. For τ*_ae_^0^*/λ*_r_^0^* approximately 1, the system displays bistability, i.e. bistability is the result of the right balance between the extent to which effector T cells are inhibited versus their effectiveness. If regulatory T cells do not inhibit effector T cells, bi-stability is not predicted by our model.

### B) Responding or not responding?

Any immune system has to be able to tolerate certain stimuli, while responding to other, such as pathogens. In the third parameter regime, in which both, stronlgy and weakly regulated states, can be attained, our model displays a dynamical behavior that enables the host's immune system to prevent autoimmunity while still allowing for effective responses against pathogens. Attaining the strongly regulated state corresponds to the immune system's tolerance to a given antigenic stimulus, while attaining the weakly regulated state corresponds to mounting a strong immune response against an antigen. Experimental data on adoptive transfer of tolerance suggest the existence of such a bi-stability (reviewed in [19]).

We investigated into which state — the strongly or the weakly regulated — the immune system described by our model falls in response to different antigenic stimuli, such as pathogens, allergens, or self-antigens. Obviously, a well-designed immune system should become tolerant in response to allergens and self-antigens, while mounting an immune response against pathogens.

We simulated the response against antigens in two different ways: by perturbing the system from a virgin state, in which there are only naïve APCs and precursor T cells, or from the strongly regulated (tolerant) state. The virgin state was perturbed by introducing a certain number of antigens, *X^0^*, which roughly corresponds to the introduction of a pathogen or an allergen that the immune system has not encountered before. The hyper-regulated state, on the other hand, was perturbed by an “inflammatory burst” (a short, but intensive upregulation of APC activation), which roughly corresponds to a spontaneous immune reaction against a self-antigen. We then examine how the immune system reacts to these perturbations, i.e. into which equilibrium state the system falls after the perturbation, and how its reaction depends on key parameters of the regulatory system.

#### Factors leading to strong primary immune responses

**Figure 4:**
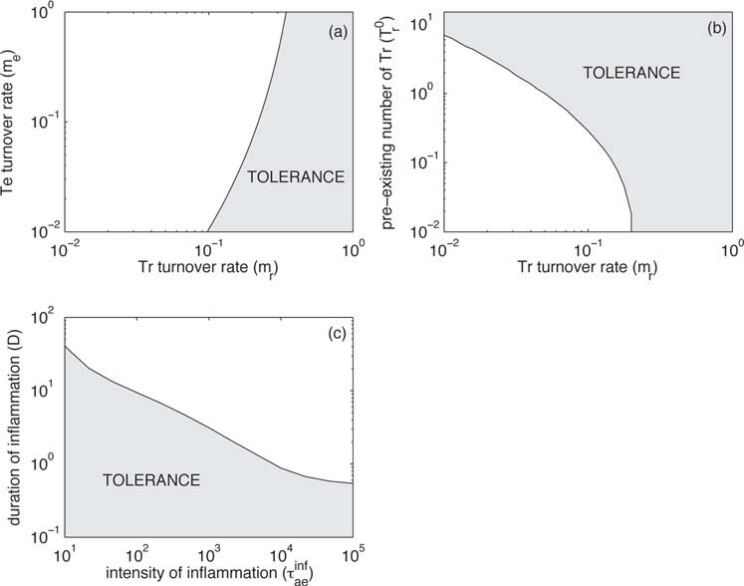
Factors leading to strong primary immune responses. Values of the parameters are as in Fig. 2c (basic: τ*_ae_^0^* = 10^4^, λ*_r_^0^* = 10^4^, τ*_r_^0^* = 10 and see Table 1) so that we are in the bi-stability region (regime 3). Initially, there are no effector T cells. At time *t* = 0, one (i.e. the maximum quantity) antigen is introduced (*X^0^* = 1): we neglect the growing phase of the antigen. Grey zones correspond to tolerance, i.e. the system falls into the strongly regulated state. White zones correspond to the development of a strong immune response, i.e. the system falls into the weakly regulated state. (a) Effect of the the effector and regulatory T cells turnover rates (*m_e_* and *m_r_*, respectively), with τ*_ap_* = 10^−2^; (b) effect of the pre-existing number of regulatory T cells (*T_r_^0^*) and the regulatory T cells turnover rate (*m_r_*), with τ*_ap_* = 10^−2^; and (c) impact of an inflammatory burst on the development of an immune response, depending on its duration (*D*) and intensity (τ*_ap_^inf^*), with τ*_ap_^rest^* = 10^−2^ and *T_r_^0^* = 1. Note that in Fig. 4a *T_r_^0^* = 0.

To investigate potential primary immune responses, we perturb an immune system from its virgin state. The relative growth rate of regulatory and effector T cells determines into which state the system falls after perturbation. Due to model assumptions, the growth rate of the effector and regulatory T cell population is inversely proportional to their turnover rates *m_e_* and *m_r_*, respectively. In Fig. 4a, we show into which state the immune system falls as a function of *m_e_* and *m_r_*. We find that the state into which the immune system falls after perturbation is more sensitive to changes in *m_r_* than to changes in *m_e_*. This is intuitive because regulatory T cells are the only force leading to de-activation of APCs, whereas APCs are activated by effector T cells as well as the antigen. Thus, the influence of effector T cell turnover on the reaction of the immune system is less pronounced.

Until now we assumed that at time *t* = 0 no regulatory T cells is present in the organism. With this assumption, we ignore, for example, the potential pre-existence of natural regulatory T cells that can exert non-specific inhibition. Many experimental studies suggest that these natural regulatory T cells are essential in preventing immunopathologies [1], [20]. Thus, the generation of adaptive regulatory T cells in response to an antigen is, on its own, not sufficient to prevent an aberrant immune response.

To model the different kinds of pre-existing regulatory cells and cytokines, we assume that at time *t* = 0 a given number of regulatory T cells (*T_r_^0^*) are present. Fig. 4b shows into which state the system falls as a function of the level of pre-existing regulatory cells, *T_r_^0^* and their turnover rate, *m_r_*. When the turnover rate of regulatory T cells is small (leading to a low growth rate of the regulatory T cells population), pre-existing regulatory T cells are necessary to maintain tolerance. The critical number of pre-existing regulatory T cells required for tolerance increases with the time required to put the adaptive regulatory response in place (i.e. decreases with *m_r_*). There is a threshold value of the turnover rate of regulatory cells beyond which pre-existing regulatory cells are not needed for tolerance (at approximately 0.2 in Fig. 4b).

#### Short-term inflammation helps to trigger a long lasting immune response

Another important factor for the long-term establishment of an immune response is inflammation. The most famous example is the use of adjuvant with vaccine. By inducing local inflammation, adjuvant helps developing a long lasting immune response. Here we model inflammation by assuming that, after the introduction of the pathogen, there is a period of time during which the activation rate of APCs by the antigen is larger. Once the inflammation period is finished the activation rate of APCs by the antigen comes back to its normal level. We denote the duration of the inflammation period by *D*, and the antigen-induced activation rate during and after the inflammation as 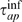 and 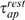, respectively. We observe that the system falls into a weakly regulated state for high 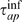 or high *D*, i.e. a strong immune response is mounted after either strong or long inflammatory episodes (Fig. 4c).

#### Overcoming strong regulation of an immune response against persistent antigens (e.g. autommunity) requires a longer inflammation

Now, we focus on persistent antigens, e.g. self-antigens that may continuously stimulate the immune system. We assume that the immune response against the persistent antigen is in the strongly regulated state (unlike for introduced antigens for which we assumed the immune system to be in a virgin state). At time *t* = 0, we then perturb the system by simulating an inflammatory episode as above.

**Figure 5:**
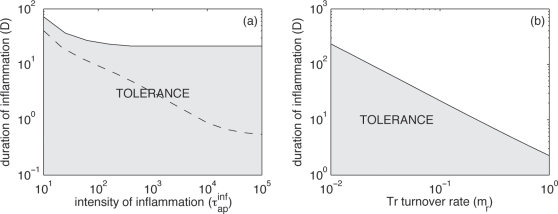
Stability of the strongly regulated equilibrium. It is characterized by the mean period of time (*D*) during which inflammation must be maintained to induce a long lasting immune response. Again values of the parameters are as in Fig. 2c (basic: τ*_ae_^0^* = 10^4^, λ*_r_^0^* = 10^4^, τ*_r_^0^* = 10 and see Table 1). (a) Effect of the duration (*D*) and intensity (τ*_ap_^inf^*) of the inflammatory burst. The threshold line obtained with the same parameters but for an introduced antigen is reported on the graph as a dashed line (with *T_r_^0^* = 1, see Fig. 4d); and (b) Effect of the duration of the inflammatory burst (*D*) and the regulatory T cells turnover rate (*m_r_*), with τ*_ap_^inf^* = 10^3^. As in Fig. 4 grey zones correspond to tolerance and white zones correspond to the development of a strong immune response. In (a) and (b), τ*_ap_^rest^* = 10^−2^.

We estimate the minimum duration of inflammation required to bring the immune system into the weakly regulated state, i.e. to trigger a strong long-lasting immune response. Qualitatively, our results are similar to those found for introduced antigens. We find the same negative relationship between the intensity of the stimulation and the minimal duration of inflammation (Fig. 5a). Interestingly tolerance is more easily achieved than for introduced antigens. In other words, chronic exposition to antigens increases the number of regulatory cells and cytokines, making tolerance harder to break.

Finally, breaking tolerance once the strongly regulated equilibrium is established requires clearing the regulatory T cell population. Unsurprisingly, large turnover rates of regulatory T cells favor the establishment of a long lasting immune response (Fig. 5b).

## Discussion

The purpose of an immune system is the defense against pathogens. A well-designed immune system, however, will require safe-guards against immune responses that are misdirected (e.g. directed against non-pathogenic antigens or self-antigens) or too strong. Experimental immunology deals with characterizing the players of the immune system (including the safe-guard mechanisms) and their molecular and cellular interactions. In addition to the characterization of the players and their interactions, however, understanding the function of an immune system requires an analysis of the dynamical properties of the interaction networks.

Viewing the immune system as a dynamical system, one can derive some desired properties a priori. For example, an immune system should have multiple stable states, such as a state of responsiveness, or a state of tolerance against an antigen. Experiments on adoptive transfer of tolerance [1], [21]–[24] show the bistable nature of the immune system. The experiments consist in the transfer of T cells from donors that are either tolerant or responsive to some antigens. Depending on the quantity and nature of the cells transferred, the recipient may mount an immune response or become tolerant. Dynamical systems analysis then allows us to investigate when certain states are attained and under which circumstances states are changed.

In this paper, we analyzed the dynamical properties of an interaction network between adaptive regulatory T cells and antigen presenting cells proposed by Powrie and Maloy [7]. We find that, for some parameters, the dynamical system representing that interaction network has two stable equilibria.

Such a bistable dynamic can be obtained with regulatory T cells through diverse interaction networks [19], [25], for example the cross-regulation model (reviewed in [26]). The cross-regulation model describes the interaction between natural regulatory T cell, the effector cell they suppress and APCs. One fundamental assumption for the occurence of bi-stabe dynamics in that model is that the growth of the natural regulatory T cells population depends on the interaction with effector cells they suppress. It is interesting to note that our model also displays the existence of two stable states as the crossregulation model although it describes the interaction of different cell types and mechanisms. As a consequence, our interpretation of the significance of the bistability is similar.

Similarly to the cross-regulation model, our model is consistent with empirical evidence (see [26] for a full review of the consistency between the cross-regulation model and empirical evidence). Our model is consistent with experiments about adoptive transfer of tolerance, which reveal the importance of the initial balance between regulatory and effector T cells. Another feature of our model is that regular exposition to tolerated antigens stimulates the production of adaptive regulatory T cells and thus maintains the system in a more tolerant state. This can explain why repeated exposure to self-antigens are necessary to maintain self-tolerance [27].

As the cross-regulation model [26], the model also yields a dual role of repeated exposition to pathogenic antigens: they can induce as well as break tolerance. Repeated exposition to benign and tolerated pathogens leads to the production of regulatory T cells, which helps to maintain tolerance. This improved tolerance has also consequences for other non-cross reacting antigens, since the production of IL-10 by adaptive regulatory T cells may exert a bystander effect on other effector cells and renders APCs more tolerant. The induction of tolerance by repeated exposition to pathogens is consistent with the hygiene hypothesis [28], [29], according to which the increase in the number of allergies that has been observed these past decades is due to the improvement of hygiene conditions. On the other hand, non-tolerated pathogens may cause strong local inflammation. This can lead to the development of an immune response against other, normally tolerated, antigens, as has been observed experimental studies [30]–[32] and linked to the adjuvant effect.

Although both our model and the cross-regulation model explain the same set of experimental observations, they also display important differences. In the cross-regulation model one fundamental assumption is the requirement for effector cells for the replication of regulatory cells. Such a factor can be IL-2, which is secreted by effector cells and is required for the growth of the natural regulatory T cell population (see [33] for a review). The cross-regulation model is consistent with observations in IL-2 deficient mice, which spontaneously develop autoimmunity [34]. However this is not in contradiction with our model, since in any case preventing the growth of the natural regulatory cells reduces the initial regulatory force, and hence can lead to autoimmunity in our model too. Another major difference between our model and the cross-regulation model is the role of resting APCs. Experimental evidence shows that the adoptive transfer of immature DCs results in an important increase in the number of IL-10 producing cells [17], [35] and helps to resolve inflammation and maintain tolerance [17], [36], which is consistent with our model. Hence it is possible that the cross-regulation model and the induction of regulatory T cells by the interaction with resting APCs are both important and complementary mechanisms for the maintenance of tolerance.

The consistency between the model proposed here and experimental results about tolerance suggest a potential role that induction of regulatory T cells by resting APCs [37], [38] in concert with adaptive regulatory T cells [39]–[41] could play in the prevention of autoimmunity. In fact, experimental studies show that both natural [1], [20] and adaptive [39]–[41] regulatory T cells are crucial for preventing autoimmune diseases. Asseman et al. [42] observed that natural regulatory T cells may control inflammatory bowel disease, but that IL-10 is mandatory for the control of the disease. Surprisingly they also observed that the transfer of natural CD4+CD25+ regulatory T cells isolated from IL-10-/- mice still inhibited the disease. These observations are easily explained by our model since experiments also show that natural regulatory T cells can induce the differentiation of precursor T cells into IL-10 producing regulatory T cells [43] and thus act as an initial regulatory force to maintain tolerance.

Finally, we would like to emphasize that, in our model, we make quite strongly simplifying assumptions about the generation of the immune response. In our model, the dynamic of both effector and regulatory T cells are reduced to simple turnover. In fact, type 1 regulatory T cells, a class of IL-10 producing adaptive regulatory T cells, can proliferate in response to IL-15 [44]. Effector cells also maintain themselves by homeostatic proliferation after their differentiation from naive/precursor cells. How tolerance is maintained in the periphery in the long-term depends on the homeostatic proliferation of the different cell types – effector T cells and adaptive and natural regulatory T cells – and cannot be addressed properly in this paper. We also neglect several important features that arise during chronic infections: the differentiation of effector T cells differentiate into memory cells, the fact that continuous exposure to antigens leads to effector T cell exhaustion [45] and differentiation into regulatory cells [46]. For these reasons, the model presented in this paper is not appropriate to study the immune response during chronic infections. However, despite these simplifying assumptions regarding the immune response, we can use this model to investigate the early immune response and in particular if, in response to a perturbation, an immune response will be mounted or not. In the model, convergence is rather fast (basically it is of the order of magnitude of the inverse of the turnover rates), so the equilibrium in which the system falls gives a good idea of how the system behaves in the few weeks that follow the perturbation.
